# 2,2′-Bipyridine–cyclo­pentane-1,2,3,4-tetra­carb­oxy­lic acid (1/1)

**DOI:** 10.1107/S1600536811025827

**Published:** 2011-07-09

**Authors:** Jian-Li Lin, Xia-Xia Guo, Wen-Xiang Huang

**Affiliations:** a Center of Applied Solid State Chemistry Research, Ningbo University, Ningbo, Zhejiang 315211, People’s Republic of China

## Abstract

The asymmetric unit of the title compound, C_10_H_8_N_2_·C_9_H_10_O_8_, contains a half-molecule of 2,2′-bipyridine and a half-molecule of 1,2,3,4-cyclopentanetetracarboxylic acid, both components being completed by crystallographic inversion symmetry. In the crystal, the mol­ecules are assembled into chains extending along [010] by O—H⋯N hydrogen bonds; adjacent chains are linked by O—H⋯O hydrogen bonds into a three-dimensional network.

## Related literature

For general background to coordination polymers, see: Bowers *et al.* (2005 ▶); Bowes *et al.* (2003 ▶). For related structures, see: Chen *et al.* (2005 ▶). 
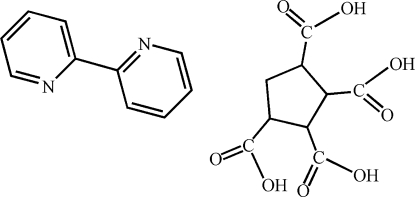


## Experimental

### 

#### Crystal data

C_10_H_8_N_2_·C_9_H_10_O_8_
*M* *_r_* = 402.35
Orthorhombic, 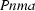

*a* = 12.942 (3) Å
*b* = 25.118 (5) Å
*c* = 5.4353 (11) Å
*V* = 1766.8 (6) Å^3^
*Z* = 4
Mo *K*α radiation
μ = 0.12 mm^−1^
*T* = 293 K
0.44 × 0.36 × 0.27 mm

#### Data collection

Rigaku R-AXIS RAPID diffractometer
Absorption correction: multi-scan (*ABSCOR*; Higashi, 1995 ▶) *T*_min_ = 0.949, *T*_max_ = 0.968
15967 measured reflections
2054 independent reflections
1499 reflections with *I* > 2σ(*I*)
*R*_int_ = 0.055

#### Refinement

*R*[*F* ^2^ > 2σ(*F* ^2^)] = 0.038
*wR*(*F* ^2^) = 0.089
*S* = 1.02
2054 reflections
141 parameters
H atoms treated by a mixture of independent and constrained refinement
Δρ_max_ = 0.19 e Å^−3^
Δρ_min_ = −0.16 e Å^−3^

### 

Data collection: *RAPID-AUTO* (Rigaku, 1998 ▶); cell refinement: *RAPID-AUTO*; data reduction: *CrystalStructure* (Rigaku/MSC, 2004 ▶); program(s) used to solve structure: *SHELXS97* (Sheldrick, 2008 ▶); program(s) used to refine structure: *SHELXL97* (Sheldrick, 2008 ▶); molecular graphics: *SHELXTL* (Sheldrick, 2008 ▶); software used to prepare material for publication: *SHELXL97*.

## Supplementary Material

Crystal structure: contains datablock(s) global, I. DOI: 10.1107/S1600536811025827/jh2304sup1.cif

Structure factors: contains datablock(s) I. DOI: 10.1107/S1600536811025827/jh2304Isup2.hkl

Supplementary material file. DOI: 10.1107/S1600536811025827/jh2304Isup3.cml

Additional supplementary materials:  crystallographic information; 3D view; checkCIF report

## Figures and Tables

**Table 1:** Hydrogen-bond geometry (Å, °)

*D*—H⋯*A*	*D*—H	H⋯*A*	*D*⋯*A*	*D*—H⋯*A*
O2—H2*B*⋯O3^i^	0.87 (2)	1.80 (2)	2.6520 (16)	165 (2)
O4—H4*A*⋯N1^ii^	0.94 (2)	1.80 (2)	2.7335 (18)	169 (2)

Symmetry codes: (i) 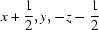; (ii) 
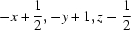
.

**Fig. 1.**
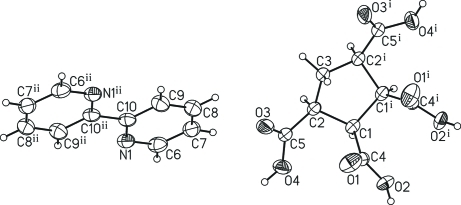
ORTEP view of the title compound, The dispalcement ellipsoids are drawn at 45% probability dispalcement ellipsoids. [Symmetry codes: (i)x, –y+1/2, z;(ii)–x, y + 1, –z.]

**Fig. 2.**
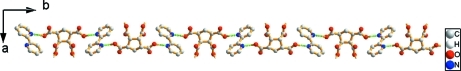
one-dimensional chain of the title cocrystal viewed along the b axis. O–H···N hydrogen bonds are shown as dashed lines.

**Fig. 3.**
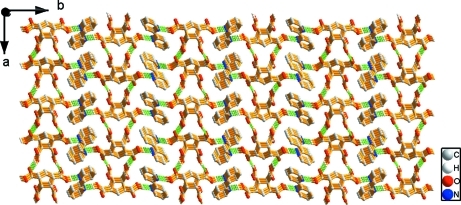
Packing diagram of the title co-crystal viewed down the c axis. O–H···N and O–H···O hydrogen bonds are shown as dashed lines.

## supplementary crystallographic information

### Comment

In hydrogen-bonded adducts of simple di–, tri– and tetracarboxylic acids with tertiary diamines, the modes of hydrogen-bonded chains are often determined by hard and soft types. Our investigation builds on the associative behavior of carboxyl and pyridine functions. In this contribution, we report the title compound with bipyridine–cyclopentanetetracarboxylic acid cocrystal.

The asymmetric unit contains one 2,2'-bipyridine molecule and one 1,2,3,4–cyclopentanetetracarboxylic acid molecule. Both bipyridine and cyclopentanetetracarboxylic acid molecules are generated *via* crystallographic 2–fold rotation axes (Fig. 1), and C3 atoms are located at the Wyckoff 4c sites. The carboxylic groups of cyclopentanetetracarboxylic acid connect with the corresponding bipyridine molecules through O4–H4A···N1^ii^ hydrogen bonds generating a one-dimensional chain along [010] (Fig. 2). In this way, the adjacent one-dimensional chains are interconnected by O2–H2B···O3^i^ hydrogen bonds to give three-dimensional network parallel to (001) (Fig. 3).

### Experimental

Under continuous stirring, a solution of 2,2'-bipyridine (0.1560 g, 1.00 mmol) in 10 ml CH_3_OH was added dropwise to an aqueous solution of 1,2,3,4–cyclopentanetetracarboxylic acid (0.1230 g, 0.50 mmol) in 10 ml H_2_O. The resulting mixture was further stirred for *ca* 30 min. After slow evaporation of the solution for one week at 35°C, colorless pillar sized crystals were obtained.

### Refinement

H atoms bonded to C atoms were palced in geometrically calculated position and were refined using a riding model, with *U*_iso_(H) = 1.2 *U*_eq_(C). H atoms attached to O atoms were found in a difference Fourier synthesis and were refined using a riding model, with the O—H distances fixed as initially found and with *U*_iso_(H) values set at 1.2 *U*eq(O).

### Crystal data

**Table:**

C_10_H_8_N_2_·C_9_H_10_O_8_	*F*(000) = 840
*M**_r_* = 402.35	*D*_x_ = 1.513 Mg m^−^^3^
Orthorhombic, *P**n**m**a*	Mo *K*α radiation, λ = 0.71073 Å
Hall symbol: -P 2ac 2n	Cell parameters from 8201 reflections
*a* = 12.942 (3) Å	θ = 3.2–27.5°
*b* = 25.118 (5) Å	µ = 0.12 mm^−^^1^
*c* = 5.4353 (11) Å	*T* = 293 K
*V* = 1766.8 (6) Å^3^	Block, white
*Z* = 4	0.44 × 0.36 × 0.27 mm

### Data collection

**Table:**

Rigaku R-AXIS RAPID diffractometer	2054 independent reflections
Radiation source: fine-focus sealed tube	1499 reflections with *I* > 2σ(*I*)
graphite	*R*_int_ = 0.055
Detector resolution: 0 pixels mm^-1^	θ_max_ = 27.4°, θ_min_ = 3.2°
ω scans	*h* = −16→16
Absorption correction: multi-scan (*ABSCOR*; Higashi, 1995)	*k* = −32→32
*T*_min_ = 0.949, *T*_max_ = 0.968	*l* = −7→7
15967 measured reflections	

### Refinement

**Table:**

Refinement on *F*^2^	Primary atom site location: structure-invariant direct methods
Least-squares matrix: full	Secondary atom site location: difference Fourier map
*R*[*F*^2^ > 2σ(*F*^2^)] = 0.038	Hydrogen site location: inferred from neighbouring sites
*wR*(*F*^2^) = 0.089	H atoms treated by a mixture of independent and constrained refinement
*S* = 1.02	*w* = 1/[σ^2^(*F*_o_^2^) + (0.034*P*)^2^ + 0.4998*P*] where *P* = (*F*_o_^2^ + 2*F*_c_^2^)/3
2054 reflections	(Δ/σ)_max_ = 0.001
141 parameters	Δρ_max_ = 0.19 e Å^−^^3^
0 restraints	Δρ_min_ = −0.16 e Å^−^^3^

### Special details

**Table:**

Geometry. All e.s.d.'s (except the e.s.d. in the dihedral angle between two l.s. planes) are estimated using the full covariance matrix. The cell e.s.d.'s are taken into account individually in the estimation of e.s.d.'s in distances, angles and torsion angles; correlations between e.s.d.'s in cell parameters are only used when they are defined by crystal symmetry. An approximate (isotropic) treatment of cell e.s.d.'s is used for estimating e.s.d.'s involving l.s. planes.
Refinement. Refinement of *F*^2^ against ALL reflections. The weighted *R*-factor *wR* and goodness of fit *S* are based on *F*^2^, conventional *R*-factors *R* are based on *F*, with *F* set to zero for negative *F*^2^. The threshold expression of *F*^2^ > σ(*F*^2^) is used only for calculating *R*-factors(gt) *etc*. and is not relevant to the choice of reflections for refinement. *R*-factors based on *F*^2^ are statistically about twice as large as those based on *F*, and *R*- factors based on ALL data will be even larger.

### Fractional atomic coordinates and isotropic or equivalent isotropic displacement parameters (Å^2^)

**Table:**

	*x*	*y*	*z*	*U*_iso_*/*U*_eq_	
C1	0.51833 (9)	0.28149 (5)	0.1032 (3)	0.0282 (3)	
H1A	0.5337	0.2944	0.2694	0.034*	
C2	0.40596 (10)	0.29744 (5)	0.0333 (3)	0.0311 (3)	
H2A	0.3672	0.3004	0.1875	0.037*	
C3	0.36186 (15)	0.2500	−0.1051 (4)	0.0354 (5)	
H3A	0.3842	0.2500	−0.2754	0.043*	
H3B	0.2869	0.2500	−0.0997	0.043*	
O1	0.58022 (9)	0.31419 (5)	−0.2864 (2)	0.0560 (4)	
O2	0.68873 (8)	0.30931 (4)	0.0286 (2)	0.0429 (3)	
C4	0.59708 (10)	0.30368 (5)	−0.0745 (3)	0.0313 (3)	
O3	0.33802 (8)	0.35651 (4)	−0.2754 (3)	0.0535 (4)	
O4	0.44673 (9)	0.38926 (4)	0.0007 (2)	0.0438 (3)	
C5	0.39410 (10)	0.34981 (6)	−0.0985 (3)	0.0341 (3)	
N1	0.07695 (9)	0.51473 (5)	0.2707 (2)	0.0392 (3)	
C6	0.14948 (12)	0.49161 (7)	0.4103 (3)	0.0456 (4)	
H6A	0.1737	0.5103	0.5463	0.055*	
C7	0.19052 (12)	0.44202 (7)	0.3654 (3)	0.0453 (4)	
H7A	0.2406	0.4276	0.4683	0.054*	
C8	0.15516 (13)	0.41452 (7)	0.1634 (3)	0.0448 (4)	
H8A	0.1816	0.3811	0.1258	0.054*	
C9	0.08006 (13)	0.43719 (6)	0.0176 (3)	0.0430 (4)	
H9A	0.0553	0.4190	−0.1193	0.052*	
C10	0.04132 (11)	0.48714 (6)	0.0747 (3)	0.0357 (3)	
H2B	0.7334 (16)	0.3216 (8)	−0.077 (4)	0.074 (7)*	
H4A	0.4380 (18)	0.4198 (10)	−0.098 (5)	0.095 (8)*	

### Atomic displacement parameters (Å^2^)

**Table:**

	*U*^11^	*U*^22^	*U*^33^	*U*^12^	*U*^13^	*U*^23^
C1	0.0233 (6)	0.0356 (7)	0.0258 (7)	−0.0014 (5)	0.0007 (6)	−0.0040 (6)
C2	0.0225 (6)	0.0321 (7)	0.0389 (8)	−0.0010 (5)	0.0033 (6)	−0.0016 (6)
C3	0.0260 (9)	0.0286 (10)	0.0517 (14)	0.000	−0.0046 (10)	0.000
O1	0.0469 (7)	0.0863 (10)	0.0350 (7)	−0.0119 (6)	0.0054 (5)	0.0090 (6)
O2	0.0249 (5)	0.0494 (7)	0.0544 (8)	−0.0087 (5)	0.0019 (5)	0.0014 (6)
C4	0.0279 (7)	0.0290 (7)	0.0371 (9)	−0.0010 (6)	0.0046 (6)	−0.0054 (6)
O3	0.0430 (6)	0.0380 (6)	0.0796 (9)	−0.0087 (5)	−0.0285 (6)	0.0102 (6)
O4	0.0467 (6)	0.0340 (6)	0.0508 (7)	−0.0104 (5)	−0.0073 (5)	−0.0026 (5)
C5	0.0236 (6)	0.0311 (7)	0.0475 (9)	−0.0015 (6)	0.0004 (7)	−0.0026 (7)
N1	0.0387 (7)	0.0378 (7)	0.0411 (8)	−0.0084 (6)	−0.0029 (6)	0.0029 (6)
C6	0.0430 (8)	0.0494 (9)	0.0443 (10)	−0.0126 (8)	−0.0101 (8)	0.0036 (8)
C7	0.0390 (8)	0.0458 (9)	0.0510 (11)	−0.0051 (7)	−0.0069 (8)	0.0125 (8)
C8	0.0467 (9)	0.0394 (9)	0.0483 (10)	−0.0018 (7)	0.0023 (8)	0.0083 (8)
C9	0.0507 (9)	0.0386 (8)	0.0396 (9)	−0.0046 (7)	−0.0054 (8)	0.0021 (7)
C10	0.0375 (7)	0.0353 (8)	0.0343 (8)	−0.0093 (6)	0.0013 (7)	0.0057 (7)

### Geometric parameters (Å, °)

**Table:**

C1—C4	1.5105 (19)	O4—C5	1.3177 (17)
C1—C2	1.5555 (18)	O4—H4A	0.94 (2)
C1—C1^i^	1.582 (3)	N1—C6	1.339 (2)
C1—H1A	0.9800	N1—C10	1.352 (2)
C2—C5	1.506 (2)	C6—C7	1.376 (2)
C2—C3	1.5202 (19)	C6—H6A	0.9300
C2—H2A	0.9800	C7—C8	1.375 (2)
C3—C2^i^	1.5202 (19)	C7—H7A	0.9300
C3—H3A	0.9700	C8—C9	1.377 (2)
C3—H3B	0.9700	C8—H8A	0.9300
O1—C4	1.2015 (19)	C9—C10	1.386 (2)
O2—C4	1.3194 (17)	C9—H9A	0.9300
O2—H2B	0.87 (2)	C10—C10^ii^	1.490 (3)
O3—C5	1.2166 (19)		
			
C4—C1—C2	112.31 (12)	O2—C4—C1	111.99 (13)
C4—C1—C1^i^	111.65 (7)	C5—O4—H4A	108.6 (14)
C2—C1—C1^i^	104.92 (7)	O3—C5—O4	121.84 (14)
C4—C1—H1A	109.3	O3—C5—C2	123.90 (13)
C2—C1—H1A	109.3	O4—C5—C2	114.19 (13)
C1^i^—C1—H1A	109.3	C6—N1—C10	117.60 (14)
C5—C2—C3	114.28 (13)	N1—C6—C7	124.23 (16)
C5—C2—C1	115.88 (11)	N1—C6—H6A	117.9
C3—C2—C1	105.67 (12)	C7—C6—H6A	117.9
C5—C2—H2A	106.8	C8—C7—C6	117.89 (16)
C3—C2—H2A	106.8	C8—C7—H7A	121.1
C1—C2—H2A	106.8	C6—C7—H7A	121.1
C2—C3—C2^i^	103.21 (17)	C7—C8—C9	119.11 (16)
C2—C3—H3A	111.1	C7—C8—H8A	120.4
C2^i^—C3—H3A	111.1	C9—C8—H8A	120.4
C2—C3—H3B	111.1	C8—C9—C10	120.02 (16)
C2^i^—C3—H3B	111.1	C8—C9—H9A	120.0
H3A—C3—H3B	109.1	C10—C9—H9A	120.0
C4—O2—H2B	110.7 (14)	N1—C10—C9	121.14 (14)
O1—C4—O2	123.15 (14)	N1—C10—C10^ii^	116.88 (17)
O1—C4—C1	124.84 (13)	C9—C10—C10^ii^	121.98 (18)

Symmetry codes: (i) *x*, −*y*+1/2, *z*; (ii) −*x*, −*y*+1, −*z*.

### Hydrogen-bond geometry (Å, °)

**Table:**

*D*—H···*A*	*D*—H	H···*A*	*D*···*A*	*D*—H···*A*
O2—H2B···O3^iii^	0.87 (2)	1.80 (2)	2.6520 (16)	165 (2)
O4—H4A···N1^iv^	0.94 (2)	1.80 (2)	2.7335 (18)	169 (2)

Symmetry codes: (iii) *x*+1/2, *y*, −*z*−1/2; (iv) −*x*+1/2, −*y*+1, *z*−1/2.
